# Isolation, Purification, and Application of Protoplasts and Transient Expression Systems in Plants

**DOI:** 10.3390/ijms242316892

**Published:** 2023-11-29

**Authors:** Kebin Chen, Jiali Chen, Xin Pi, Li-Jun Huang, Ning Li

**Affiliations:** 1 Key Laboratory of Cultivation and Protection for Non-Wood Forest Trees, Ministry of Education, Central South University of Forestry and Technology, Changsha 410004, China; 2 Key Laboratory of Forest Bio-Resources and Integrated Pest Management for Higher Education in Hunan Province, Central South University of Forestry and Technology, Changsha 410004, China

**Keywords:** protoplasts, transient transformation, gene expression, plants, application

## Abstract

Protoplasts, derived from plant cells, exhibit remarkable totipotency and hold significant value across a wide spectrum of biological and biotechnological applications. These versatile applications encompass protein subcellular localization and interaction analysis, gene expression regulation, functional characterization, gene editing techniques, and single-cell sequencing. Protoplasts’ usability stems from their inherent accessibility and their ability to efficiently incorporate exogenous genes. In this review, we provide a comprehensive overview, including details on isolation procedures and influencing factors, purification and viability assessment methodologies, and the utilization of the protoplast transient expression system. The aim is to provide a comprehensive overview of current applications and offer valuable insights into protoplast isolation and the establishment of transient expression systems in a diverse range of plant species, thereby serving as a valuable resource for the plant science community.

## 1. Introduction

Protoplasts, intriguing cellular entities defined as the protoplasmic mass devoid of a cell wall and enveloped by the cell membrane, constitute essential building blocks within the intricate tapestry of cellular biology [1,2]. Their fascinating characteristics and versatile applications have piqued the interest of plant research in various fields. These unique structures house a wealth of genetic information, endowing them with the remarkable ability to engage in processes such as reproduction, differentiation, and, under the right conditions, regeneration into fully formed plants. Their lack of a cell wall is a pivotal feature, as it renders them exceptionally receptive to the uptake of exogenous genetic material, making them coveted subjects for genetic transformation experiments. A prominent application of protoplasts lies in the realm of plant protoplast fusion technology, which has the power to overcome the barriers of affinity between distant plant species, thereby facilitating genetic recombination. Through this innovative approach, scientists can harness the genetic diversity of different plant species, potentially leading to the development of novel plant varieties with desirable traits.

Moreover, the utility of protoplasts extends to the realm of protoplast culture, where these cellular entities can be coaxed into generating healing tissues. These tissues, in turn, serve as the foundation for the regeneration of complete plant bodies. This process holds immense promise for crop improvement, tissue culture, and plant breeding efforts [3]. In essence, the versatility of plant protoplasts as research tools and biotechnological agents is unparalleled. They serve as cornerstones for plant transgenic technology, enabling the introduction of foreign genes into plant cells for the development of genetically modified organisms (GMOs). Additionally, they play a pivotal role in somatic cell hybridization, facilitating the creation of novel hybrid plants with advantageous traits.

In view of the diverse and promising applications of plant protoplasts, this review attempts to provide a comprehensive overview of the evolving isolation methods and their versatile applications. Nowadays, it has become a trend to use protoplasts for in planta expression to avoid the discrepancy of in vitro expression in certain model plants, which leads to the instability of research results. We present a comprehensive analysis and review of the current, up-to-date advances in protoplast isolation and transient transformation systems, with the aim of advocating the use of protoplast systems in a wide range of plants.

## 2. Protoplast Isolation

### 2.1. Plant Tissues for Protoplast Isolation

The choice of materials for isolating plant cell protoplasts plays a pivotal role in determining both the yield and viability of the resulting protoplasts [4]. Commonly employed materials for protoplast isolation include young leaves [5], petals [6], roots [7], callus [8], and suspension cultures [9]. And it has been shown that vigorously growing and younger tissues used as isolation materials tend to yield protoplasts with higher yields and vitality [10]. Comparative studies have indicated that protoplasts extracted from petals outperform those derived from young leaves [11]. Furthermore, under identical enzymatic conditions, protoplasts isolated from suspension cultures exhibit the highest yield and vigor, followed by sterile seedling leaves, with guaiac tissues yielding the lowest [12]. It is important to note that the optimal choice of isolation materials and enzymatic digestion time can vary among different plant species, necessitating preliminary testing to determine the most suitable materials. Table 1 provides an overview of the isolation materials, enzymatic solutions, protoplast quantities, and protoplast activities of different plant species.

### 2.2. Methods of Protoplast Separation and Isolation

Protoplast isolation methods primarily fall into two categories: mechanical separation and enzymatic digestion. The mechanical method involves placing cells in a hypertonic solution, which induces the separation of the plasma wall, leading to the exudation of cellular material and the subsequent division of plant tissue, resulting in the release of protoplasts [23]. However, this technique is limited to specific tissues and is typically only effective with larger, highly vascularized cells found in storage tissues such as onion bulb scales, carrots, and beetroot. A drawback of this method is that substances released from crushed cells can significantly reduce the viability of other protoplasts.

The enzymatic isolation of protoplasts represents a widely employed technique in protoplast extraction [58]. It operates on the principle of employing enzymes, including cellulase, hemicellulose, dissociative enzymes, and pectinase, to facilitate the degradation of the plant cell wall, leading to the acquisition of protoplasts [59,60]. Specifically, pectinase and dissociative enzymes primarily target the breakdown of pectin molecules, promoting the separation of adjacent cells. In the process of protoplast isolation, it is common practice to blend cellulase and pectinase [61]. Initially, pectinase is utilized to degrade pectin, after which the cell wall undergoes further degradation under the sustained action of cellulase, ultimately liberating the protoplasts. This enzymatic separation method yields a substantial quantity of protoplasts characterized by their structural integrity and exhibits a broad range of applications.

### 2.3. Pretreatment of Enzymatically Isolated Plant Material

Pretreatment of plant material is a crucial step aimed at facilitating the penetration of enzymes into the plant cell wall, ultimately enhancing the yield of isolated protoplasts. Several pretreatment methods are employed for this purpose, each tailored to optimize the isolation process. Dark incubation involves subjecting the plants designated for protoplast isolation to a period of incubation in darkness [62]. This process typically spans several hours and serves to augment the viability of the resultant protoplasts. By shielding the plant material from light, dark incubation minimizes the risk of photodamage during the isolation procedure. Preplasmic wall separation treatment [10] entails immersing the enzymatically treated material in a cell protoplast washing solution, a mannitol solution of a specific concentration, or a combination thereof. This immersion may occur under static conditions or with controlled rotation for a predetermined duration. The objective is to expedite the plasmic wall separation process of chloroplasts [23], thereby facilitating the subsequent isolation of protoplasts. This treatment strategy leads to a notable increase in the yield of protoplasts [42]. Low-temperature treatment involves subjecting the plant material to a cold environment for a defined period, typically within a refrigerator set at 4 °C. This treatment is often combined with dark incubation [63]. The primary aim is to mitigate the potential damage to protoplasts while concurrently augmenting both the quantity and viability of the isolated protoplasts. These pretreatment methods play a pivotal role in optimizing the isolation of plant protoplasts, enhancing the efficiency of enzyme action, and ultimately improving the overall yield and quality of the isolated protoplasts.

### 2.4. Factors Influencing the Preparation of Protoplasts

The preparation of protoplasts is subject to several critical factors that influence the efficiency of the process. Mechanical methods are, in general, restricted to tissues and cells characterized by a high degree of vascularization and, consequently, are associated with notably low protoplast yields. The enzymatic method, on the other hand, is profoundly influenced by several key factors, including the choice of enzyme, enzyme concentration, osmotic pressure, enzymatic digestion temperature, enzymatic digestion duration, and purification methodology [64]. Enzymes employed for cell wall degradation primarily consist of cellulase, hemicellulose, and pectinase, chosen based on the predominant components of the cell wall. Cellulase, a complex enzyme, exhibits the capability to hydrolyze high-molecular-weight cellulose molecules into lower-molecular-weight cellulose molecules, ultimately breaking them down into simple monosaccharides. Hemicellulose collectively refers to enzymes proficient in hemicellulose hydrolysis [65]. Pectinase effectively catalyzes the hydrolysis of pectin into β-galacturonide, facilitating its detachment from individual cells. The selection of dissociation enzymes is contingent upon the specific plant species or distinct tissues within the same plant, necessitating the establishment of customized dissociation protocols [66]. Owing to material requirements and the yield considerations associated with isolated protoplasts, the enzymatic approach remains the predominant method for protoplast isolation [60,64].

Indeed, numerous factors, such as enzyme selection, concentration, osmotic conditions, temperature, and duration of enzymatic digestion, are instrumental in the efficient isolation of protoplasts. The choice of dissociation enzymes must be tailored to the plant species or tissue type, and as a result, enzymatic methods continue to be the preferred approach for protoplast isolation due to their versatility and effectiveness.

## 3. Purification of Protoplasts

Upon enzymatic digestion of plant material using an enzymatic solution, the resultant mixture comprises a heterogeneous blend of biological materials. This mixture includes not only intact and metabolically active protoplasts but also residual enzymatic solutions, materials that remain undigested, inactivated and damaged protoplasts, as well as a substantial quantity of cellular debris and organelles. The presence of these various components can significantly affect the physiological state and various applications of protoplasts, making purification a vital and intricate step in the process. The ultimate aim of purification is to obtain a purified protoplast population with high viability and integrity, suitable for downstream experiments and applications. The initial phase of purification is focused on removing large debris materials, such as cellular tissue and undigested cell clusters. It is achieved by passing the mixture through a sieve with a specific mesh size, typically ranging from 40 to 100 μm [25]. This filtration process effectively eliminates these larger, unwanted components, leaving behind a mixture that contains both protoplasts and smaller debris particles.

Currently, there are several methods employed for the purification of protoplasts, each with its own unique advantages and considerations. These methods include sedimentation, floating, and interfacial techniques. The sedimentation method, or centrifugal precipitation method, is based on the principle of specific gravity. After initial filtration to remove large debris, the mixture is subjected to centrifugation. During centrifugation, protoplasts, due to their specific gravity, will sediment to the bottom of the centrifuge tube in a solution with a controlled osmotic pressure. Although this method may cause a certain degree of mechanical damage to protoplasts during the centrifugation process, it is known for yielding a large number of protoplasts. The floating method exploits the specific gravity differences between protoplasts and hypertonic sucrose solutions. When subjected to centrifugation, protoplasts rise to the upper liquid surface, while tissue residues and other debris sediment to the bottom of the centrifuge tube. The floating method is renowned for providing relatively high purity and completeness of protoplasts. However, one trade-off associated with the floating method is that it often results in a relatively lower yield of protoplasts [10]. The interface method, alternatively referred to as the interfacial method [67], is a technique that relies on the creation of two different solutions with distinct osmotic concentrations. This choice of solutions results in a specific density range wherein protoplasts are concentrated, allowing for effective purification.

Therefore, the purification of protoplasts is a critical and intricate aspect of the isolation process, essential for obtaining a high-quality protoplast population suitable for a wide range of research applications. The choice of purification method depends on specific research goals and experimental requirements, with each method offering unique advantages and considerations. As such, the selection of an appropriate purification method is pivotal in ensuring the success of subsequent experiments and investigations involving isolated protoplasts.

## 4. Protoplast Yield and Viability Assay

Throughout the intricate process of protoplast isolation, several variables, including enzymatic activity, operational procedures, and inherent factors, can potentially lead to the damage and breakage of protoplasts. Consequently, the quantity and quality of protoplasts obtained during the crucial phase are of paramount importance. Their characteristics profoundly impact the efficiency and success of subsequent protoplast transformation procedures, directly influencing the subsequent transformation efficiency [66].

The accurate quantification of protoplast yield is a fundamental step in the isolation process. Currently, the most prevalent method for assessing protoplast yield involves the use of a hemocytometer. Through a meticulous counting process, the protoplast yield can be reliably estimated. Equally important is the assessment of protoplast viability, which provides insights into the overall health and vitality of the isolated protoplast population. Various staining methods are employed for this purpose, with fluorescein diacetate (FDA) staining being one of the most widely used techniques. FDA is a nonfluorescent compound that possesses the unique ability to permeate the cell membrane. Within living cells, FDA undergoes enzymatic degradation, resulting in the production of yellow–green fluorescent compounds. Conversely, nonviable protoplasts lack the necessary enzymatic activity for FDA degradation and, consequently, do not emit fluorescence. Fluorescence microscopy is utilized to meticulously observe the ratio of fluorescent protoplasts to the total number of protoplasts present, allowing for the precise determination of protoplast viability [64].

The assessment of both protoplast yield and viability is a pivotal aspect of protoplast isolation. Hemocytometers provide a reliable means of quantifying yield, while staining techniques such as FDA staining are favored for assessing protoplast viability. The accurate evaluation of these parameters is imperative to ensure the success of subsequent protoplast transformation experiments and to achieve robust research outcomes in the fields of protoplast-based biotechnology and plant science.

## 5. Protoplasmic Transient Gene Expression System

The protoplast transient expression system is a powerful biotechnological tool designed for the efficient introduction and expression of target genes within plant protoplasts over a short duration [68]. This system is distinguished by its ability to facilitate rapid gene expression analysis with high throughput. Leveraging reporter genes, the gene transient expression system has found extensive utility in diverse areas of gene function analysis, including investigations into target gene expression, subcellular protein localization, promoter and protein activity assessment, protein–protein interactions, and other related studies. The protoplast transient expression system encompasses the process of introducing exogenous genes into plant protoplasts and integrating them into the protoplast genome for subsequent expression via specific pathways or technologies, employing protoplasts as the receptive host.

Several protoplast transient expression techniques have been developed, including the PEG-mediated method [69], the electroshock method [70], the Agrobacterium-mediated method [71], and the liposome-mediated method [72]. PEG-mediated transformation exploits polyethylene glycol (PEG) to bind to cell membranes, disturbing the phospholipid bilayer and destabilizing the cell surface potential [35]. Consequently, this facilitates the introduction of plasmid or T-DNA into the protoplast [73]. Electroshock transformation involves the application of a brief, high-voltage pulse to stimulate plant cell membranes temporarily. This stimulation results in the transient formation of small pore channels through which exogenous DNA molecules can swiftly enter the protoplast [73]. In the Agrobacterium co-transformation method, exogenous genes are introduced into protoplasts through co-culturing Agrobacterium with the protoplasts, leading to the generation of transgenic plants [74]. Liposome-mediated transformation entails the encapsulation of DNA within liposomes, followed by contact and fusion with protoplasts, ultimately enabling the transfer of exogenous genes into the protoplasts [75]. Among these methods, the PEG-mediated approach currently stands as the most widely adopted due to its simplicity, convenience, high frequency of induced fusion, lack of species-specific requirements, and minimal dependence on specialized instrumentation [76]. The protoplast transient expression system represents a versatile technology in plant biotechnology and research. It enables rapid and high-throughput gene expression analysis within plant protoplasts, with various transformation techniques available for researchers to choose from based on their specific experimental needs and preferences.

## 6. Applications of Protoplasts and Their Transient Transformation Systems

**Figure 1:**
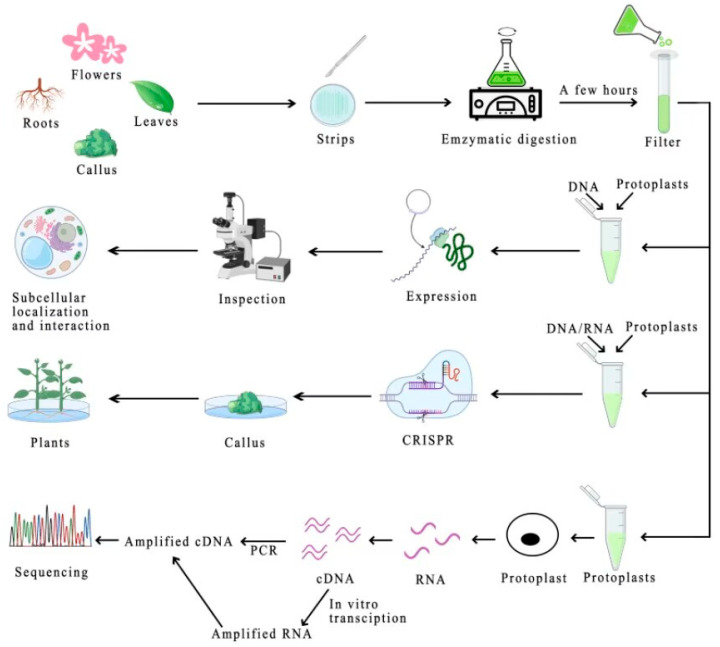
Schematic workflow of isolation and different applications of plant protoplasts. The broad pathway for obtaining protoplasts involves treating the material, enzymatically digesting the material, and then purifying it by filtration. Subcellular localization of proteins and examination of protein interactions using purified plant protoplasts. Through CRISPR/Cas9-mediated gene editing technology, targeted mutations in the plant genome are induced using a plant protoplast transient expression system, and then, by tissue culture of the gene-edited protoplasts, plants with the desired properties can be obtained. Single-cell sequencing using protoplasts is also possible, avoiding the difficulties of using tissue samples that do not yield information on the heterogeneity between different cells or that are difficult to be routinely sequenced.

The development and refinement of protoplast isolation techniques, coupled with the establishment of transient transformation systems, have paved the way for a diverse array of applications in the fields of plant science, genetics, molecular biology, and biotechnology. Protoplasts offer a compelling platform for the elucidation of fundamental plant biology, including gene expression regulation, protein localization, and interactions, as well as the functional characterization of genes. Beyond these fundamental aspects, protoplasts have found pivotal roles in cutting-edge gene editing technologies and single-cell RNA sequencing (Figure 1).

### 6.1. Protein Subcellular Localization and Interaction Analysis

**Figure 2:**
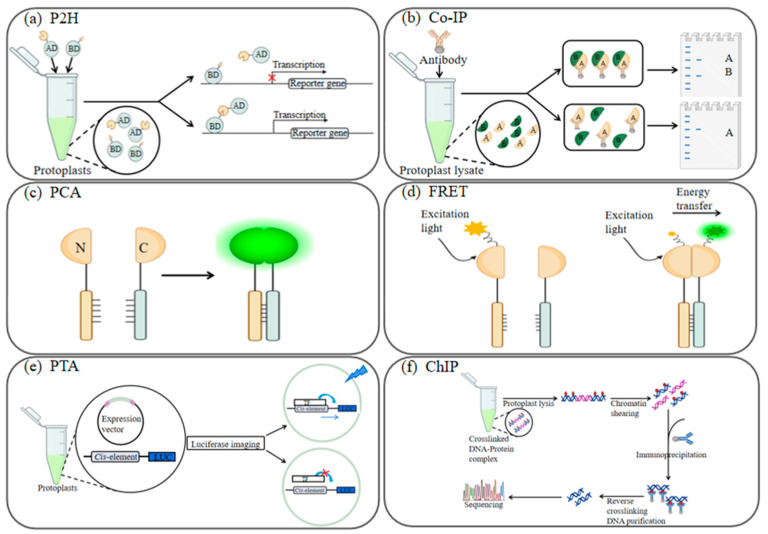
Schematic representation of protein-protein interactions investigated via plant protoplasts. (**a**) In the protoplast two-hybrid (P2H) system, the two investigated proteins to be tested were constructed with the BD and AD structural domains to build a fusion plasmid, respectively, and the constructed two plasmids were transferred into the same yeast cell for expression; if there is no interaction between the two proteins, the reporter gene will not be transcribed and expressed; if there is interaction between the two proteins, the reporter gene will be transcribed; (**b**) In co-immunoprecipitation (co-IP), an antibody to protein A (Anti-HA) is added to protoplast lysates containing protein A and protein B. If proteins A and B interact, the two proteins will be observed by Western blotting (WB). If there is no interaction between proteins A and B, the Western blot detects only protein A; (**c**) In protein complementation assay (PCA), such as the bimolecular fluorescence complementation assay (BiFC), a fluorescent protein polypeptide chain gets severed and a target protein attaches to each polypeptide, resulting in a target protein comprising a C-(carboxy-terminal) and another target protein containing an N-(amino-terminal). If the two target proteins interact, their interaction leads to binding between the N- and C-fragments, resulting in fluorescence restoration; (**d**) Fluorescence resonance energy transfer (FRET) is a spectroscopic analysis method that examines fluorescent substances by non-radiative energy transfer that occurs between them. When two proteins interact and bind to separate fluorescent molecules, the excited molecule can transfer its energy to the other molecule, resulting in alterations to the fluorescence intensity and lifetime of the donor molecule; (**e**) Schematic overview of the protoplast trans-activation (PTA) system. Expression vectors were obtained by BP reaction and LR reaction, and the vectors and Luciferase reporter were co-transfected into protoplasts. If the promoter can be activated by the transcription factor (TF), then fluorescence can be detected using the instrument. Otherwise, there will not be any fluorescence; (**f**) Chromatin immunoprecipitation (ChIP). Proteins in living cells are transiently bound to the associated chromatin and the DNA-protein complex (chromatin-protein) is then sheared into DNA fragments by enzymatic digestion. DNA fragments cross-linked with formaldehyde-associated proteins are selectively immunoprecipitated from cellular debris using specific protein-specific antibodies. After reversal of cross-linking, the relevant DNA fragments are purified and sequenced.

Subcellular localization, which pertains to the precise intracellular positioning of cellular components and structures, is a pivotal aspect of understanding cellular function and molecular interactions (Figure 2). Within this context, the term subcellular encompasses a wide range of cellular constituents, including the nucleus, chloroplasts, mitochondria, and various enzymatic and proteinaceous entities that partake in cell metabolism [77]. Protoplasts, owing to their absence of a cell wall, offer an advantageous platform for the efficient transfection of multiple DNA structures concurrently. This expedites experimental timelines and yields rapid results, making the establishment of transient expression systems utilizing protoplasts as the host a favored approach for studying protein subcellular localization and interactions. For example, Li et al. examined millet protoplasts and identified SiARDP, SiAREB1, and SiAREB2, which were found to be localized within the nucleus [78]. Co-localization was confirmed, with AHL22 serving as a positive control protein for this nuclear localization. Hillwig et al. investigated ribonuclease T2 family genes, constructing the *RNS2* gene vector and transiently expressing it in Arabidopsis protoplasts [79]. Experimental results revealed that the *RNS2* gene-expressed proteins were localized in the endoplasmic reticulum, subsequently diffusing to the cytoplasm and vesicles for expression. Djemal et al. delved into resistance-regulated genes in wheat using the tobacco protoplast system, determining that the AP2/ERF-type transcription factor HvSHN1 predominantly localizes to the nucleus [80]. Wang et al., in their investigation of key enzymes for flavonoid biosynthesis in grapevine suspension cell protoplasts, established that VvCHS, VvCHI, VvUFGT, and VvANR are primarily localized in the cytoplasm and nucleus [81]. Xu et al. explored the subcellular localization of GhPIN1 in cotton cotyledon protoplasts using a transient expression system, revealing its expression in the cell membrane [82]. While studying the tobacco transient expression system, Liu et al. identified RIN13 as being expressed in the nucleus [83]. Transient expression of RIN13 in *Tobacco benthamiana* leaves accelerated leaf senescence and cell death. Localization studies have also been reported in other subcellular organelles and compartments, such as chloroplasts [84], mitochondria [85], vesicles [86], and cell membranes [87].

Protoplast transient expression technology, when coupled with bimolecular fluorescence complementation (BiFC) [88], provides a highly intuitive approach for visualizing protein interactions [89]. Lin et al., in their study of the oil tea petal protoplast transient expression system, observed intracellular expression of CoSVP in petal protoplasts, while CoAP1 was exclusively expressed in the nucleus. Furthermore, they elucidated an interaction between CoSVP and CoAP1, shedding light on the regulatory role of CoAP1 in the subcellular localization of CoSVP and underscoring their co-expression in the nucleus [19]. Ren et al. established that CsAP3 and CsPI proteins are localized within the nucleus in a transient expression system using orchid protoplasts [11]. Additionally, they discerned a significant direct interplay between these two proteins. The application of protoplast transient expression technology has facilitated detailed investigations into subcellular localization and protein interactions. These studies provide invaluable insights into the intricate workings of plant cells, offering a deeper understanding of cellular function and molecular dynamics.

### 6.2. Gene Expression Regulation and Functional Assays

Protoplasts have emerged as versatile tools for investigating gene expression regulation and function, which allows various facets of gene activity, including upstream signal regulation, downstream gene regulation, and the validation of gene function through expression studies. Protoplasts provide an ideal platform for scrutinizing the transcriptional expression of genes (Figure 2). This enables the intricate regulatory mechanisms governing both the genes being introduced into protoplasts and their downstream targets. Moreover, gene expression analysis offers a means of confirming the functional relevance of specific genes. Kim et al. identified the transcription factor OsWRKY71 and conducted a transient expression analysis in maize chloroplast protoplasts. Their findings revealed the predominant nuclear localization of OsWRKY71, with some presence in the cytoplasm [90]. This transcription factor played a positive role in enhancing cold tolerance by regulating downstream target genes. Nakashima et al. elucidated the subcellular localization and function of OsNAC6 in a transient expression system using rice protoplasts. OsNAC6 was found to be nuclear-localized and functioned as a transcriptional activator in response to various abiotic and biotic stresses, thereby enhancing plant stress tolerance [91]. Chen et al. validated the significant cell death-inducing activity of two genes, *NLP1* and *NIS1*, in a transient expression system established in melon (*Cucumis melo*) leaves [92]. Sakuma et al. investigated the drought stress tolerance enhancement achieved by DREB2A in plants through a transient expression study [93]. In studies related to starch synthesis regulation [94], researchers screened and investigated the gene *ZmbZIP91*, a key regulator of starch synthesis. The function of this gene was verified by transferring it into protoplasts and subsequently assessing its impact on the expression of starch synthesis genes. Protoplast-based transient expression systems serve as important tools for unraveling the intricacies of gene expression regulation and function. Most recently, we reconstructed a JA (jasmonic acid) signaling pathway in protoplasts, encompassing JA perception, signal transduction, and transcriptional regulation, which not only reaffirmed previous discoveries but also enabled the exploration of uncharacterized details within the JA signaling pathway [95]. The signaling pathway reconstruction in protoplasts offers a comprehensive strategy for employing synthetic biology methodologies to investigate and engineer cell signaling networks, thereby creating novel cellular functions within plant systems [96]. These studies contribute to our understanding of the molecular mechanisms governing gene activity, facilitating advancements in various fields, from stress tolerance enhancement to signaling pathway regulation in plants.

### 6.3. Gene Editing Advancements in Protoplasts

Protoplasts have emerged as invaluable resources in the realm of genetic engineering, offering a promising avenue for enhancing plant genetic improvement. Amid the challenges of chimeric plant emergence in conventional genetic transformation, protoplasts stand as a potent solution [97]. Their unique attribute of lacking cell walls allows for the precise introduction of specific genes through the direct uptake of exogenous genetic material. Consequently, transgenic plants derived from single cells can be developed, effectively mitigating chimerism and elevating the success rate of transgenesis. In tissue culture, individual protoplasts can be employed to culture and regenerate entire plants. This approach holds immense potential in breeding programs, where protoplast fusion can simplify distant hybridization challenges, paving the way for the development of high-yield novel plant varieties. The combination of transient expression techniques with traditional genetic breeding allows for the precise modification of plant traits [98]. This includes enhancing genes related to drought resistance, cold tolerance, insect resistance, and facilitating the improvement and selection of plant varieties.

Plant genome editing has witnessed a paradigm shift with the advent of advanced tools. The CRISPR/Cas system, zinc finger nucleases, and transcription activator-like effector nucleases are among the prominent genome editing tools. Presently, the CRISPR/Cas system reigns supreme as the most widely adopted genome editing tool. Malnoy et al. successfully isolated protoplasts from grapes and apples and harnessed the CRISPR/Cas system to achieve precise, targeted mutagenesis [99]. Specifically, they targeted the grape locus *MLO-7* and the apple loci *DIPM-1*, *DIPM-2*, and *DIPM-4*, resulting in effective gene modifications. Mariette et al. embarked on a groundbreaking endeavor to perform whole gene knockouts in tetraploid potatoes through transient expression in protoplasts [100]. Their work harnessed the CRISPR/Cas9 system, specifically targeting the *GBSS* gene, and successfully obtained mutations in four alleles. Subsequently, these mutations were employed in breeding programs to establish potato genotypes tailored for further studies. With the CRISPR/Cas system at the forefront, plant biologists have made significant strides in enhancing plant traits, paving the way for the development of genetically improved and resilient plant varieties.

### 6.4. Protoplasts Empowering Single-Cell RNA Sequencing

Single-cell RNA sequencing (scRNA-seq) has emerged as a transformative technology that operates at the level of individual cells, addressing issues related to tissue samples’ limitations in capturing cellular heterogeneity and the challenges posed by insufficient sample sizes for conventional sequencing approaches. Leveraging the potential of protoplasts has further propelled the capabilities of scRNA-seq, enabling comprehensive exploration of single-cell transcriptomes. Application of protoplast-based scRNA-seq in plant biology enables the study of gene expression in plant cells at the single-cell level, which is valuable for understanding plant development, responses to environmental cues, and crop improvement. Xie et al. introduced a pioneering approach by developing a protocol for the preparation of rice chloroplast protoplasts and conducting scRNA-seq on these individual cells [101]. This innovative endeavor led to the screening and identification of Os01g0934800 and Os01g0949900 as targets of OsNAC78, shedding light on their regulatory roles. Li et al. embarked on a groundbreaking exploration of wood formation at a single-cell level through scRNA-seq [102]. Their efforts resulted in the establishment of high-resolution expression profiles, offering invaluable insights into the intricate processes underpinning wood formation. Single-cell RNA sequencing has ushered in a new era of transcriptome analysis, transforming our capacity to dissect transcriptional heterogeneity among cells. By harnessing the power of protoplasts, we can delve deep into the transcriptomes of individual cells, transcending the limitations of bulk tissue sampling. This innovative approach not only expands our understanding of cellular diversity but also paves the way for breakthroughs in diverse fields, from plant biology to developmental studies and beyond.

## 7. Summary Remarks and Future Perspectives

The isolation and culture of plant protoplasts are intricate processes influenced by various factors. These methods can significantly differ between plant species and tissue types, as summarized in Table 1. To achieve successful protoplast isolation, meticulous control of enzyme digestion time and mannitol concentration is essential. The choice of enzymes, including cellulases, dissociating enzymes, pectinases, and others, should be tailored to the specific plant and tissue characteristics. Purification of protoplasts requires careful consideration of the method used, with attention to avoiding excessively high centrifugation speeds and harsh oscillations. The entire protoplast separation process should prioritize a gentle and slow approach. During protoplast culture, stringent adherence to operational standards and precautions against contamination are imperative. Additionally, the selection of culture medium, hormones, and culture techniques should be subjected to rigorous screening and repeated experimentation. It is important to note that there is no one-size-fits-all protoplast culture method, necessitating iterative trials based on the unique characteristics of the plant under investigation.

The ability to swiftly and efficiently generate substantial experimental data using transient transformation systems is invaluable in today’s research landscape. While protoplast technology has found widespread application across various fields, not all plant species can yield abundant, highly active, and high-quality protoplasts suitable for transient transformation and related experiments. Moreover, only a limited number of plant species currently have the capability for targeted gene editing through protoplast regeneration technology.

Hence, the future of plant protoplast research will focus on the investigation and optimization of protoplast isolation and regeneration systems. The ultimate goal is to establish efficient and stable protoplast isolation and regeneration protocols that can be applied across a broad spectrum of plant species. As technology continues to evolve, advancements in these areas will open up new avenues for plant research, genetic improvement, and biotechnology applications.

## Figures and Tables

**Table 1:** Optimization and comparison of protoplast isolation methods for various plants.

Plant Species	Material	Enzymes	Protoplasts Yield	References
*Albizia julibrissin*	Leaf (in vitro)	1.50% C + 1.00% P	7.77 × 10^5^/g FW 94%	[13]
*Albizia julibrissin*	Callus	2.00% C + 1.00% P	6.92 × 10^5^/g FW 92%	[13]
*Ananas comosus*	Leaf (in vitro)	1.50% C + 0.50% M	6.5 × 10^5^/g FW 51.0%	[14]
*Arabidopsis thaliana*	14-day seedlings	1.00% C + 1.00% M	>5 × 10^6^/g FW	[15]
*Brassica oleracea*	Leaf	2.00% C + 0.10% P	6.00 × 10^7^/g FW 95.0%	[16]
*Catalpa bungei*	Leaf (in vitro)	3.00% C + 2.00% M	1 × 10^6^/g FW 90%	[17]
*Calibrachoa elegans*	Leaf (in vitro)	2.00% C + 0.60% M	0.4~1.7 × 10^6^/g FW	[18]
*Camellia Oleifera*	Flower petal	3.00% C + 1.00% M	1.42 × 10^7^/g FW 88.69%	[19]
*Camellia Oleifera*	Young leaf (in vitro)	1.50% C + 0.50% M + 0.25% S	3.50 × 10^7^/g FW 90.90%	[20]
*Cannabis sativa*	Young leaf	1.50% C + 0.40% M + 1.00% P	9.7 × 10^6^/g FW	[21]
*Chirita pumila*	Young leaf	1.00% C + 0.50% M + 0.25% P	6.83 × 10^5^/g FW 92.97%	[22]
*Chrysanthemum morifolium*	Leaf (in vitro)	1.50% C + 0.50% M	6.32 × 10^5^/g FW 91.70%	[23]
*Cucumis sativus*	Leaf (in vitro)	1.50% C + 0.40% M	6.0~7.0 × 10^6^/g FW 90.0%	[24]
*Cymbidium sinense*	Flower petal	1.20% C + 0.60% M	3.50 × 10^7^/g FW 94.21%	[11]
*Cymbidium sinense*	Flower pedicel	1.20% C + 0.60% M	5.3 × 10^6^/g FW 90.3%	[25]
*Cymbidium sinense*	Young leaf	1.20% C + 0.60% M	3.3 × 10^6^/g FW 91.3%	[25]
*Cymbidium sinense*	Leaf base	1.20% C + 0.60% M	2.5 × 10^7^/g FW 92.1%	[25]
*Cymbidium sinense*	Root tip	1.20% C + 0.60% M	7.8 × 10^5^/g FW 89.3%	[25]
*Dendrobium catenatum*	Leaf	1.20% C + 0.60% M	8.2 × 10^6^/g FW 91.1%	[25]
*Echinacea augustifolia*	Callus	2.00% C +1.00% P +0.50% H	5.0 × 10^5^/g FW	[26]
*Ginkgo biloba*	Leaf	2.00% C + 0.2% P+ 1.5% M	5.39 × 10^6^/g FW 80.23%	[27]
*Ginkgo biloba*	Leaf	2.00% C +0.25% P	1.0 × 10^6^/g FW 80.0%	[28]
*Gossypium hirsutum*	Callus	1.50% C + 1.50% P + 1.50%/0.50% M + 0.50% H	3.3 × 10^6^/g FW 97%	[29]
*Gossypium hirsutum*	Young leaf	1.50% C + 0.40% M	>1.00 × 10^6^/g FW >90%	[30]
*Gossypium hirsutum*	Taproot	1.50% C + 0.75% M	3.55 × 10^5^/g FW 93.3%	[31]
*Hevea brasiliensis*	Leaf	1.50% C + 0.30% M	18.6 × 10^7^/g FW 97%	[32]
*Jasminum sambac*	Callus	1.50% C + 0.40% M + 0.80% P	2.38 × 10^7^/g FW 88%	[33]
*Liriodendron × sinoamericanum*	Leaf (in vitro)	1.50% C + 0.50% M + 0.10% P	1.2 × 10^7^/g FW 97.0%	[34]
*Manihot esculenta*	Leaf (in vitro)	1.60% C + 0.80% M	4.4 × 10^7^/g FW 92.6%	[35]
*Medicago sativa*	Legumes root	1.50% C + 2.00% M	1.0 × 10^6^/g FW >90.0%	[36]
*Nicotiana benthamiana*	Leaf (in vitro)	1.00% C + 0.50% M	4~5 × 10^6^/g FW	[37]
*Oryza sativa*	Stem and sheath	1.50% C + 0.75% M	1.0 × 10^7^/g FW >95%	[38]
*Oryza sativa*	Leaf base	1.20% C + 0.60% M	4.3 × 10^7^/g FW	[25]
*Oryza sativa*	Leaf	1.20% C + 0.60% M	No viable protoplast	[25]
*Petunia hybrida*	Leaf (in vitro)	2.00% C + 0.60% M	0.3~2.0 × 10^6^/g FW	[18]
*Phalaenopsis aphrodite*	Flower petals	1.00% C + 0.25% M	1.9 × 10^5^/g FW 90.9%	[39]
*Phalaenopsis aphrodite*	Leaf (in vitro)	1.00% C + 0.70% M	5.9 × 10^6^/g FW 57.9%	[40]
*Phalaenopsis aphrodite*	Leaf (in vitro)	2.00% C + 1.00% M	1.1 × 10^6^/g FW 83.8%	[41]
*Phalaenopsis equestris*	Leaf base	1.20% C + 0.60% M	1.8 × 10^7^/g FW 92.8%	[25]
*Phaseolus vulgaris*	Leaf	1.50% C + 0.37% M	3.0 × 10^5^/g FW	[42]
*Phaseolus vulgaris*	Flower petals	1.50% C + 0.37% M	2.0 × 10^5^/g FW	[42]
*Phaseolus vulgaris*	Hypocotyl and root	2.00% C + 0.30% M + 4.00% H	2.0 × 10^5^/g FW	[42]
*Phaseolus vulgaris*	Nodule	1.00% C + 0.30% M + 4.00% H	1.0 × 10^5^/g FW	[42]
*Populus przewalskii*	Leaf (in vitro)	2.00% C + 0.50% P	1.0 × 10^8^/g FW >82.0%	[43]
*Populus przewalskii*	Leaf	3.00% C + 0.80% P	1.0 × 10^7^/g FW >90.0%	[44]
*Prunus avium*	Cell suspension	1.00% C + 0.50% P	4.3 × 10^6^/g FW 84.1%	[45]
*Ricinus communis*	Leaf	1.50% C + 0.40% M	6.1 × 10^6^/g FW 85%	[46]
*Saccharum officinarum*	Young leaf base	2.00% C + 0.50% M	12.6 × 10^7^/g FW 80.19%	[47]
*Solanum melongena*	Leaf	1.25% C + 0.40% M	1.2 × 10^7^/g FW 96.0%	[48]
*Torenia fournieri*	Leaf	1.50% C + 0.50% M	6.0~7.0 × 10^5^/g FW	[49]
*Triticum aestivum*	Leaf	1.00% C + 0.25% M	7.3 × 10^6^/g FW 95.0%	[50]
*Uncaria rhynchophylla*	Leaf	1.25% C + 0.6% M	1.5 × 10^7^/g FW >90%	[51]
*Vernicia fordii*	Mature leaf	1.50% C + 1.00% M	7.21 × 10^6^/g FW 93.19%	[52]
*Vernicia fordii*	Young leaf (in vitro)	2.00% C + 1.00% M	7.08 × 10^6^/g FW 95.06%	[52]
*Vitis vinifera*	Cell suspension	2.00% C + 1.00% M	3~4 × 10^7^/g FW >95%	[53]
*Vitis vinifera*	Leaf (in vitro)	1.50% C + 0.40% M	3.3 × 10^6^/g FW 96.0%	[54]
*Zea mays*	Yellow leaf bases	1.50% C + 0.50% P	1.0~5.0 × 10^6^/g FW 80~90%	[55]
*Zea mays*	Leaf	0.10% C + 0.01% M	1.8~1.9 × 10^7^/g FW 95.0%	[56]
*Zea mays*	Leaf	1.20% C + 0.60% M	0.7 × 10^7^/g FW 89.2%	[25]
*Zea mays*	Leaf base	1.20% C + 0.60% M	3.2 × 10^7^/g FW 94.3%	[25]
*Zea mays*	Endosperm	1.00% C + 0.75% M	2.43 × 10^6^/g FW >80%	[57]

C, cellulase R-10; H, hemicellulose; M, macerozyme R-10; P, pectinase; S, snailase.

## Data Availability

Not applicable.
